# Risk of Secondary Malignancies After Pelvic Radiation: A Population-based Analysis

**DOI:** 10.1016/j.euros.2024.02.013

**Published:** 2024-03-23

**Authors:** Connor McPartland, Andrew Salib, Joshua Banks, James R. Mark, Costas D. Lallas, Edouard J. Trabulsi, Leonard G. Gomella, Hanan Goldberg, Benjamin Leiby, Robert Den, Thenappan Chandrasekar

**Affiliations:** a Department of Urology, Sidney Kimmel Medical College, Thomas Jefferson University, Philadelphia, PA, USA; b Department of Urology, Lewis Katz School of Medicine, Temple University, Philadelphia, PA, USA; c Division of Biostatistics, Sidney Kimmel Medical College, Thomas Jefferson University, Philadelphia, PA, USA; d Department of Urology, Einstein Healthcare Network, Philadelphia, PA, USA; e Department of Urology, SUNY Upstate Medical University, Syracuse, New York, NY, USA; f Department of Radiation Oncology, Sidney Kimmel Medical College, Thomas Jefferson University, Philadelphia, PA, USA; g Department of Urology, University of California, Davis, Sacramento, CA, USA

**Keywords:** Radiation therapy, Pelvic cancer, Second cancer, Second malignancy, Surveillance, Epidemiology, and End Results program

## Abstract

**Background and objective:**

Radiation therapy has increasingly been used in the management of pelvic malignancies. However, the use of radiation continues to pose a risk of a secondary malignancy to its recipients. This study investigates the risk of secondary malignancy development following radiation for primary pelvic malignancies.

**Methods:**

A retrospective cohort review of the Surveillance, Epidemiology, and End Results database from 1975 to 2016 was performed. Primary pelvic malignancies were subdivided based on the receipt of radiation, and secondary malignancies were stratified as pelvic or nonpelvic to investigate the local effect of radiation.

**Key findings and limitations:**

A total of 2 102 192 patients were analyzed (1 189 108 with prostate, 315 026 with bladder, 88 809 with cervical, 249 535 with uterine, and 259 714 with rectal/anal cancer). The incidence rate (defined as cases per 1000 person years) of any secondary malignancies (including but not limited to secondary pelvic malignancies) was higher in radiation patients than in nonradiation patients (incidence rate ratio [IRR] 1.04, confidence interval [CI] 1.03-1.05), with significantly greater rates noted in radiation patients with prostate (IRR 1.22, CI 1.21-1.24), uterine (IRR 1.34), and cervical (IRR 1.80, CI 1.72-1.88) cancer. While the overall incidence rate of any secondary pelvic malignancy was lower in radiation patients (IRR 0.79, CI 0.78-0.81), a greater incidence was still noted in the same cohorts including radiation patients with prostate (IRR 1.42, CI 1.39-1.45), uterine (IRR 1.15, CI 1.08-1.21), and cervical (IRR 1.72, CI 1.59-1.86) cancer.

**Conclusions and clinical implications:**

Except for localized cervical cancer, when put in the context of median overall survival, the impact of radiation likely does not carry enough weight to change practice patterns. Radiation for pelvic malignancies increases the risk for several secondary malignancies, and more specifically, secondary pelvic malignancies, but with a relatively low absolute risk of secondary malignancies, the benefits of radiation warrant continued use for most pelvic malignancies. Practice changes should be considered for radiation utilization in malignancies with excellent cancer-specific survival such as cervical cancer.

**Patient summary:**

The use of radiation for the management of pelvic malignancies induces a risk of secondary malignancies to its recipients. However, the absolute risk being low, the benefits of radiation warrant its continued use, and a change in practice patterns is unlikely.

## Take Home Message

Radiation for primary pelvic malignancies increases the risk of secondary malignancy development. However, outside of malignancies, with excellent cancer-specific survival, the impact of radiation on latency between primary and secondary malignancies is likely not enough to change practice patterns.

## 1 Introduction

Radiation therapy (RT) has emerged as an effective treatment modality in cancer treatment. With many cancers being sensitive to radiation, RT is currently used in the treatment of approximately 50% of all patients with cancer [1]. Patients receiving RT benefit from noninvasive targeting of cancer with minimal damage to surrounding healthy tissue [2]. RT has been shown to provide equivalent oncological control to other therapies, such as surgery, while remaining less invasive [3].

As multimodal treatments have evolved, RT has increasingly been utilized in the management of pelvic malignancies, either as primary treatment or in combination with surgical resection and/or chemotherapy [4], [5], [6]. Improvements in RT techniques have allowed for the delivery of more focused radiation, resulting in reduced morbidity and better patient outcomes for those receiving RT for pelvic cancer [7]. Despite these changes, RT continues to pose risk to its recipients. One significant adverse effect of RT is the development of a secondary malignancy within the radiated field [8], [9], [10]. The risk of secondary malignancy development in those receiving RT for primary pelvic malignancies has been investigated thoroughly for single primary malignancies such as prostate cancer (PCa) [8]. However, the literature lacks a comprehensive assessment of the risk for RT-induced secondary malignancies, and more specifically, RT-induced secondary pelvic malignancies, among all patients receiving RT for primary pelvic malignancies.

In this study, we examine the rate of any secondary malignancies following RT for primary pelvic malignancies, with a specific emphasis on secondary pelvic malignancies. Using the Surveillance, Epidemiology, and End Results (SEER) database, we highlight the risk of secondary malignancies among different primary pelvic malignancies treated with RT.

## 2 Patients and methods

### 2.1 Patient selection

Using the SEER database, which reports cancer-specific outcomes from specific geographic areas representing 28% of the US population, we first identified all patients diagnosed with five prespecified pelvic malignancies as their primary tumors from 1975 to 2016. Based on SEER coding, the primary pelvic malignancies were classified according to the primary tumor site and histology into five categories: bladder (BCa), cervical (CCa), PCa, rectal/anal (ARC), and uterine (UCa) cancer. We then utilized an internal SEER mechanism to identify all secondary malignancies linked to each patient in our initial cohorts; using their internal patient identification, all secondary malignancies were sequentially linked to individual patients (ranging from zero to eight unique secondary malignancies).

### 2.2 Variable selection and secondary malignancy classification

Demographic variables of interest included age at diagnosis, gender, race, insurance, marital status, and region based on the SEER registry. Utilizing prior literature [11], a county-level socioeconomic measure was created.

For primary malignancies, clinical variables of interest included tumor-node-metastasis (TNM), SEER summary stage (localized, regional, and distant) [11], and survival. Radiation receipt was documented in patients who underwent any form of RT targeting the primary malignancy, including brachytherapy, as well as in patients who received radiation before, during, and/or after surgery.

For each secondary malignancy, clinical variables of interest included TNM, SEER stage, and survival. Latency period was also identified by calculating the difference between the year of diagnosis for the primary malignancy and the year of diagnosis for the corresponding secondary malignancy.

### 2.3 Statistical analysis

Descriptive statistics for demographic variable comparisons were performed by the Student *t* test for continuous variables and the chi-square test for categorical variables. For each primary disease site, demographics were compared with and without stratification by radiation receipt. Overall survival (OS) was calculated as median OS (months) and 5-yr OS (%), stratified by SEER summary stage (localized, regional, and distant) [11]. Data from the American Cancer Society (ACS) [12], [13] were reported in a similar fashion to provide external validation.

First, we compared the incidence rates of any secondary malignancies in radiated (RT) patients with those in nonradiated (non-RT) patients, independent of stage. All RT and non-RT patients were included regardless of whether or not their clinical course included alternative or supplemental treatment modalities. The incidence rates of secondary pelvic malignancies were then analyzed to determine the local effect of RT on pelvic malignancy risk. The incidence rate ratio (IRR) of any secondary malignancies and, more specifically, secondary pelvic malignancies was calculated between RT and non-RT patients, and stratified by primary site. The *p* values were calculated using a *t* test based on Student *t* distribution. Through SAS V9.4 software [14], the cumulative incidence (CI) of all secondary malignancies and pelvic malignancies was then stratified by primary disease site, RT receipt, time frame (5, 10, and 15 yr), and SEER stage, with death being a competing risk in the calculation of CI. To better characterize the temporal impact of radiation receipt on the development of second malignancies to OS, latency period was compared between RT and non-RT patients, stratified by primary disease site and SEER stage.

## 3 Results

### 3.1 Demographics, OS, and receipt of radiation

A total of 2 102 192 patients were examined (1 189 108 with PCa, 315 026 BCa, 249 535 UCa, 259 714 ARC, and 88 809 CCa; Table 1). Table 2 documents the median OS (months) and 5-yr OS (%) based on primary malignancy and SEER stage, alongside data from the ACS [12], [13]. Supplementary Table 1 illustrates patient demographics stratified by primary disease and receipt of RT.

**Table 1:** Patient demographics stratified by disease site alone

Variable	Cervical cancer	Prostate cancer	Uterine cancer	Bladder cancer	Rectal/anal cancer
Total	88 419	1 187 995	249 345	314 293	259 350
Sex					
Male	NA	NA	NA	235 843 (75.0)	143 536 (55.3)
Female	NA	NA	NA	78 450 (25.0)	115 814 (44.7)
Age range (yr)					
20-29	6039 (6.8)	39 (0.0)	1334 (0.5)	1068 (0.3)	1585 (0.6)
30-39	19 446 (22.0)	548 (0.05)	8202 (3.3)	3803 (1.2)	7492 (2.9)
40-49	21 969 (24.9)	28 340 (2.4)	25 444 (10.2)	14 587 (4.6)	26 584 (10.3)
50-59	16 827 (19.0)	211 841 (17.8)	67 342 (27.0)	45 085 (14.3)	60 699 (23.4)
60-69	12 351 (14.0)	441 449 (37.2)	77 433 (31.1)	83 667 (26.6)	68 031 (26.2)
70-79	7385 (8.4)	364 939 (30.7)	47 117 (18.9)	95 295 (30.3)	57 509 (22.2)
80+	4402 (5.0)	140 839 (11.9)	22 381 (9.0)	70 788 (22.5)	37 450 (14.4)
Marital status					
Unknown	5207 (5.9)	135 859 (11.4)	11 463 (4.6)	19 031 (6.1)	14 467 (5.6)
Single	20 282 (22.9)	105 409 (8.9)	39 455 (15.8)	30 819 (9.8)	36 481 (14.1)
Married	39 105 (44.2)	791 016 (66.6)	128 445 (51.5)	191 476 (60.9)	142 330 (54.9)
Separated, divorced	12 761 (14.4)	78 400 (6.6)	256 659 (10.3)	24 684 (7.9)	25 838 (10.0)
Widowed	10 945 (12.4)	76 553 (6.4)	43 949 (17.6)	48 072 (15.3)	39 950 (15.4)
Unmarried or domestic partner	119 (0.1)	758 (0.1)	282 (0.1)	211 (0.1)	284 (0.1)
Stage					
Localized	43 017(48.7)	600 402 (50.5)	172 340 (69.1)	225 879 (71.9)	111 122 (42.9)
Regional	30 574(34.6)	65 421 (5.5)	41 229 (16.5)	56 395 (17.9)	87 542 (33.8)
Distant	9386 (10.6)	47 960 (4.0)	23 919 (9.6)	12 161 (3.9)	42 027 (16.2)
Unknown/unstaged	5442 (6.2)	474 212 (39.9)	11 765 (4.7)	19 858 (6.3)	18 659 (7.2)
Chemotherapy					
No/unknown	60 808 (68.9)	1 178 272 (99.2)	215 857 (86.6)	266 232 (84.7)	266 232 (84.7)
Yes	27 531 (31.1)	9723 (0.8)	33 396 (13.4)	48 061 (15.3)	48 061 (15.3)
Race					
Unknown	842(1.0)	25 404 (2.1)	1597 (0.6)	3016 (1.0)	2118 (1.0)
Hispanic	16 514 (18.7)	87 483 (7.4)	21 914 (8.8)	15 603 (5.0)	22 193 (8.6)
American Indian	847 (1.0)	4205 (0.4)	1364 (0.6)	855 (0.3)	1641 (0.6)
Asian	7742 (8.8)	54 863 (4.6)	17 067 (6.9)	11 847 (3.8)	21 033 (8.1)
Black	12 529 (14.2)	166 090 (14.0)	21 402 (8.6)	16 183 (5.2)	24 103 (9.3)
White	49 945 (56.5)	849 943 (71.5)	185 909 (74.6)	266 789 (84.9)	188 262 (72.6)
Year of diagnosis					
1975-1984	11 602 (13.1)	64 750 (5.5)	28 429 (11.4)	31 337 (10.0)	29 877 (11.5)
1985-1994	13 218 (15.0)	152 582 (12.9)	29 103 (11.7)	41 101 (13.10)	34 895 (13.5)
1995-2004	25 558 (28.9)	368 422 (31.0)	61 424 (24.6)	87 206 (27.8)	71 315 (27.5)
2005-2016	38 041 (43.0)	602 241 (50.7)	130 297 (52.3)	154 649 (49.2)	123 263 (47.5)
Radiation therapy receipt					
Yes	45 736 (51.7)	386 737 (32.6)	70 913 (28.5)	18 061 (5.7)	98 366 (37.9)
No	42 683 (48.3)	801 258 (67.4)	178 340 (71.5)	296 232 (94.3)	160 984 (62.1)

NA = not available.

**Table 2:** Median overall survival stratified by primary disease site and SEER stage

Primary malignancy	Extent of disease	Median overall survival (mo)	5-yr overall survival (%)	ACS statistics (5-yr relative survival), %
Bladder	Localized	130 (129-130)	73.02	70
	Regional	25 (25-26)	34.98	38
	Distant	5 (5-5)	4.53	6
Cervical	Localized	378 (373-386)	87.79	92
	Regional	65 (63-68)	51.12	58
	Distant	10 (10-11)	15.03	18
Prostate	Localized	189 (188-190)	87.41	99
	Regional	216 (212-221)	89.82	99
	Distant	25 (24-25)	23.63	31
Rectal/anal	Localized	150 (148-151)	73.53	91
	Regional	72 (71-73)	54.71	72
	Distant	13 (12-13)	9.69	15
Uterine	Localized	239 (239-241)	86.76	95
	Regional	113 (110-117)	61.16	69
	Distant	14 (14-14)	22.24	18

ACS = American Cancer Society; SEER = Surveillance, Epidemiology, and End Results.

### 3.2 Incidence rates of malignancies stratified by primary disease site and receipt of RT

The incidence rate of any secondary malignancies was higher in RT patients than in non-RT patients (22.34% vs 21.49%, IRR 1.04, *p* < 0.001), particularly in patients with PCa (25.57% vs 20.88%, IRR 1.22, *p* < 0.001), UCa (15.26% vs 13.48%, IRR 1.34, *p* < 0.001), and CCa (15.85% vs 8.84%, IRR 1.80, *p* < 0.0001; Table 3).

**Table 3:** Incidence rates of malignancy stratified by primary disease site and receipt of radiation therapy

Primary malignancy type	Total number of patients	Any secondary malignancy					Secondary pelvic malignancy				
		Incidence rate RT (cases per 1000 person years)	Incidence rate non-RT (cases per 1000 person years)	IRR	CI	*p* value	Incidence rate RT (cases per 1000 person years)	Incidence rate non-RT (cases per 1000 person years)	IRR	CI	*p* value
All	2 099 152	22.34	21.49	1.04	1.03–1.05	<0.001	4.01	5.05	0.79	0.78–0.81	<0.001
Bladder	314 293	35.06	33.38	1.05	1.00–1.10	0.036	11.92	14.01	0.85	0.79–0.92	<0.001
Cervical	88 419	15.85	8.84	1.80	1.72–1.88	<0.001	4.51	2.62	1.72	1.59–1.86	<0.001
Prostate	1 187 745	25.57	20.88	1.22	1.21–1.24	<0.001	3.81	2.68	1.42	1.39–1.45	<0.001
Rectal/anal	259 350	19.03	20.19	0.94	0.92–0.97	<0.001	5.37	6.71	0.80	0.77–0.83	<0.001
Uterine	249 345	15.26	13.48	1.34	1.11–1.16	<0.001	2.87	2.50	1.15	1.08–1.21	<0.001

CI = cumulative incidence; IRR = incidence rate ratio; RT = Radiation therapy.

In analyzing secondary pelvic malignancies, the incidence rate of any secondary pelvic malignancy was lower in RT patients than in non-RT patients (4.01% vs 5.05%, IRR 0.79, *p* < 0.001). However, incidence rates remained greater in RT patients with PCa (3.81% vs 2.68%, IRR 1.42, *p* < 0.001), UCa (2.87% vs 2.50%, IRR 1.15, *p* < 0.001), and CCa (4.51% vs 2.62%, IRR 1.72, *p* < 0.001).

### 3.3 Distribution of secondary pelvic malignancies

**Fig. 1:**
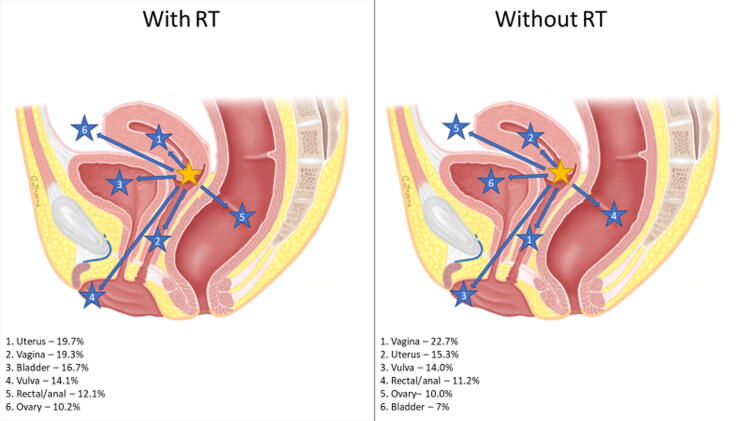

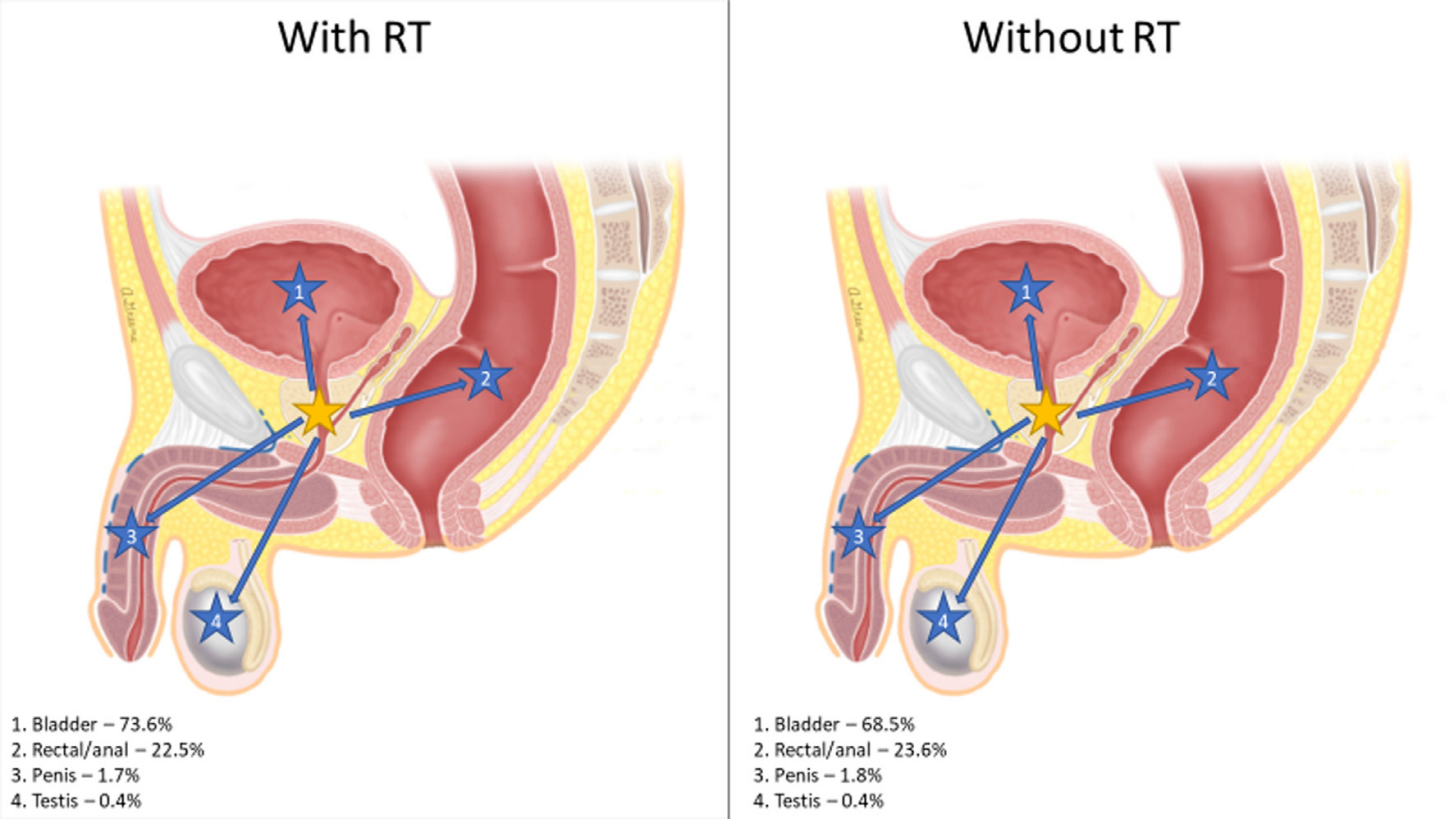

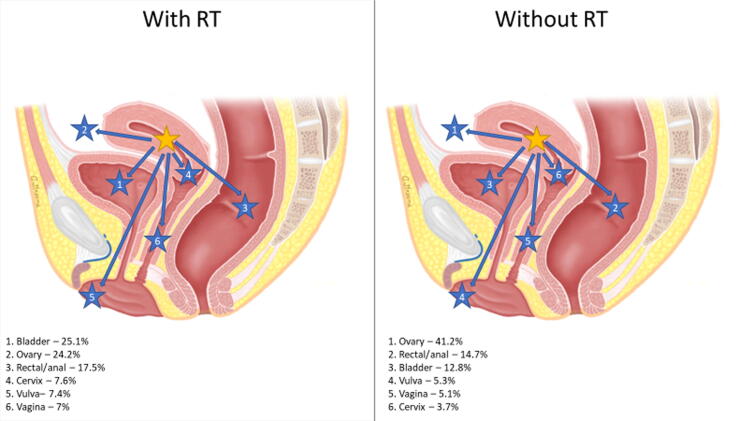

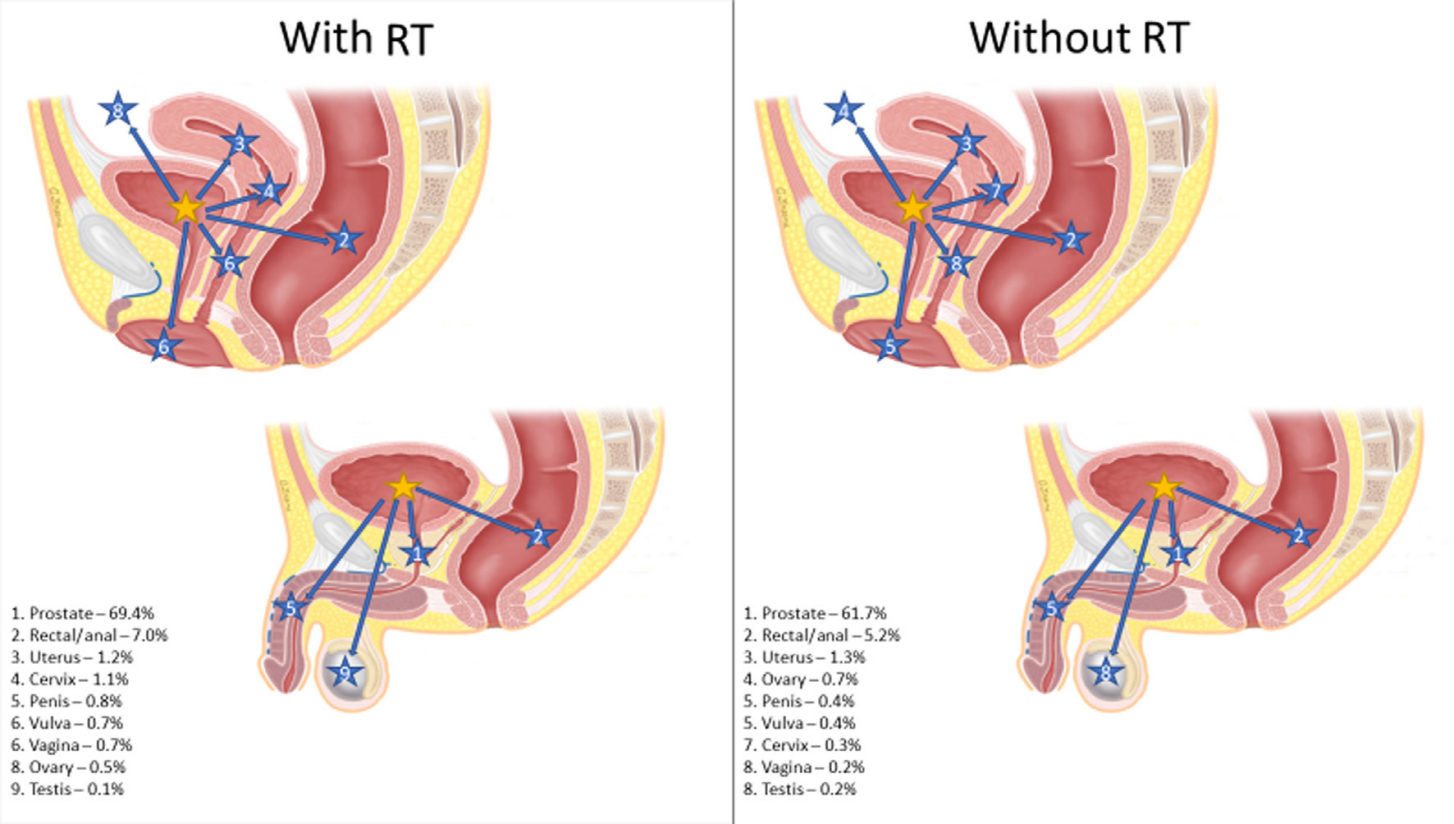

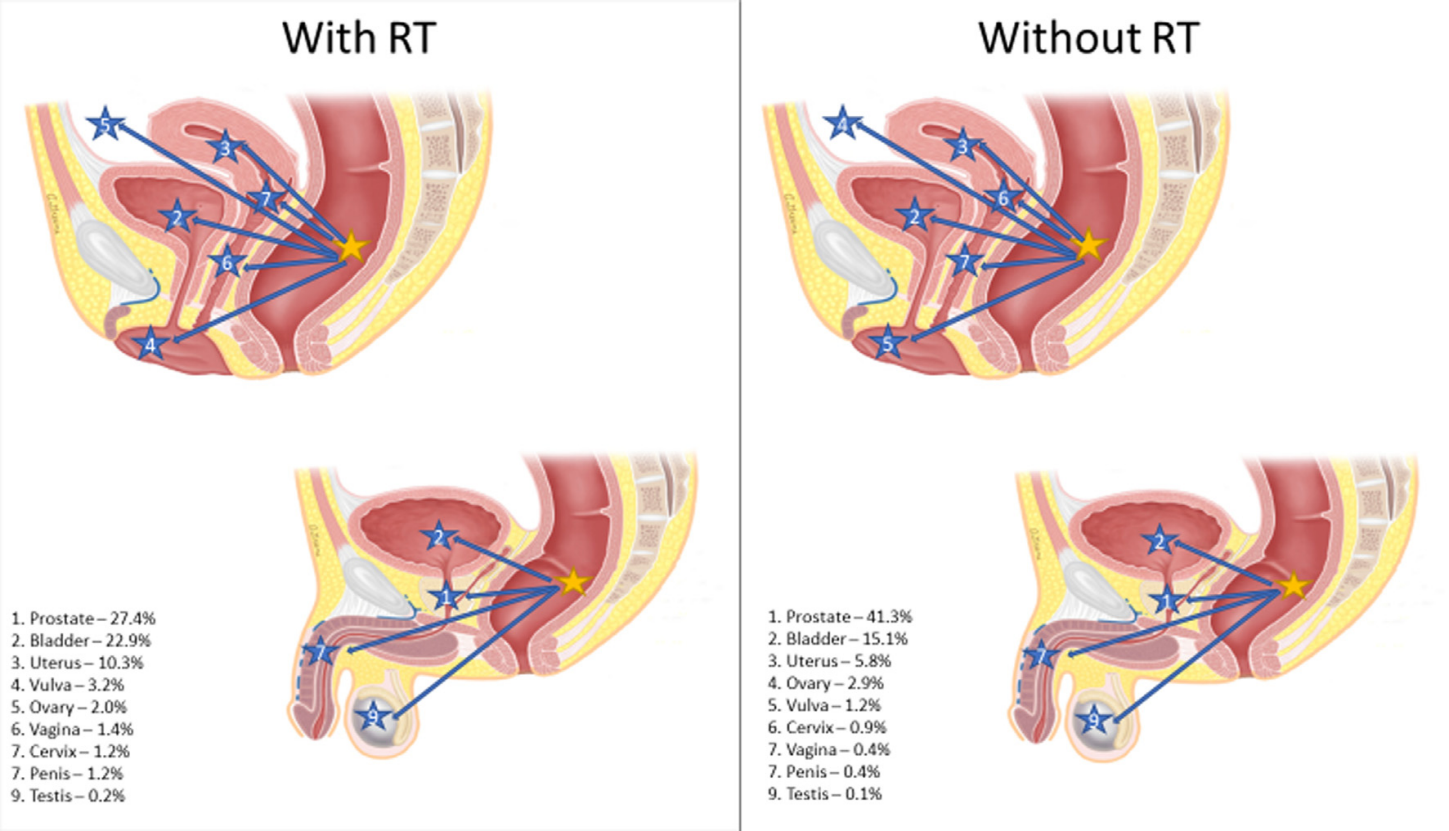
Distribution of secondary pelvic malignancies by primary disease site and relative rates impacted by RT receipt. Primary malignancy anatomical location was indicated by a yellow star, and secondary pelvic malignancies were indicated by corresponding arrows with numbered blue stars. Percentages represent the incidence of a specific secondary malignancy relative to the incidence of all secondary pelvic malignancies for a specific primary malignancy that was treated with or without RT. (A) Primary cervical cancer (female only). (B) Primary prostate cancer (male only). (C) Primary uterine cancer (female only). (D) Primary bladder cancer (need male and female). (E) Primary rectal/anal cancer (need male and female). RT = radiation therapy.

Rates of secondary pelvic malignancies were stratified by the primary disease site, secondary disease site(s), and receipt of RT. Figure 1 highlights the distribution of secondary pelvic malignancies with or without RT utilization based on the primary disease site.

### 3.4 CI stratified by primary site, SEER stage, time frame, and RT

To identify populations at a significant risk of secondary malignancies and secondary pelvic malignancies, CI was calculated in all patients stratified by primary disease site, SEER stage, RT receipt, and time frame (5, 10, and 15 yr). Results are summarized in Supplementary Table 2 (any secondary malignancy) and Supplementary Table 3 (secondary pelvic malignancies).

### 3.5 Secondary malignancy latency

**Fig. 2:**
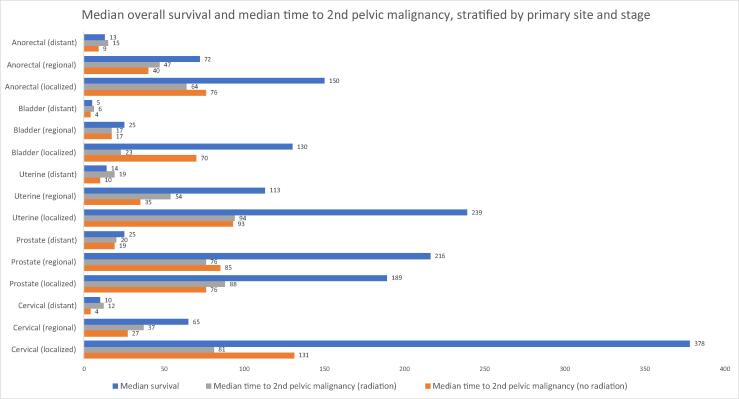
Median time to secondary pelvic malignancy relative to median overall survival, stratified by primary disease site and SEER stage. SEER = Surveillance, Epidemiology, and End Results.

To determine the clinical impact of the second pelvic malignancy, the median OS and median time between secondary pelvic malignancy diagnosis and primary malignancy diagnosis were stratified by primary disease site, RT, and SEER stage (Supplementary Table 4 and Fig. 2).

## 4 Discussion

RT is an important treatment modality in the management of pelvic malignancies [15], [16], [17]. However, RT has local side effects due to radiation injury to nearby organs [18], [19], [20]. One adverse effect of RT, which can have a significant impact on patient’s survival, is the development of a secondary malignancy. Since patients with an initial cancer diagnosis often have a higher incidence of subsequent cancer diagnosis, further elucidation of the factors that impact secondary malignancy development is necessary [19]. In this study, we examine the local effect of RT on the development of secondary pelvic malignancies.

Using the SEER database, we developed one of the largest cohorts of pelvic malignancies to ever be analyzed. Compared with other databases, specifically the cohort analyzed annually by the ACS (Table 2), our patient population experienced similar median OS and 5-yr survival rates when stratified by primary malignancy site and SEER stage, thereby validating our patient selection. In our study, as illustrated via Supplementary Table 1, >29% received RT for the management of a primary pelvic malignancy.

RT patients experienced a greater incidence of secondary malignancies in three primary sites (CCa, PCa, and UCa; Table 3), with the highest rate in CCa patients (IRR 1.80). In a subanalysis of the incidence of specifically secondary pelvic malignancies overall, RT patients were less likely to develop a secondary pelvic malignancy (IRR 0.79, CI 0.78-0.81, *p* < 0.001) than non-RT patients. However, the incidence of secondary pelvic malignancies in CCa, PCa, and UCa was greater in RT than in non-RT patients. As illustrated in Figure 1, RT had a significant impact on the distribution of secondary pelvic malignancies. Our results are consistent with other studies that showed an increase in secondary pelvic malignancies after RT [21], [22]. Secondary pelvic malignancies stratified based on SEER stage, RT receipt, and primary site illustrated significant differences in the CI of malignancies (Supplementary Table 3). The incidence of secondary pelvic malignancies in patients with localized PCa at 10 and 15 yr as well as regional PCa at 15 yr was significantly greater in RT patients. However, for all BCa patients and patients with localized ARC, RT was associated with a lower CI of secondary pelvic malignancies across all time frames. Additionally, for patients with secondary pelvic malignancies classified as distant on SEER staging, there was no difference in CI between RT and non-RT patients. Considering these findings, RT receipt is associated with variable effects across different malignancies, which may be related to the timing of RT in the treatment algorithm and competing risks of cancer-specific survival.

For PCa, RT is used as a primary treatment or as adjuvant/salvage treatment after prostatectomy [21]. The risk of secondary pelvic malignancies after PCa radiation has been well studied with a specific focus on secondary BCa [22]. Nieder et al [22] used SEER to evaluate secondary malignancy rates in RT versus radical prostatectomy (RP) patients. The study showed an increased incidence of secondary BCa and rectal cancer in patients undergoing external beam radiation therapy (EBRT), brachytherapy, or a combination of EBRT and brachytherapy when compared with RP patients [22]. These findings validate our results of an increased risk of secondary pelvic malignancies in PCa patients receiving RT.

RT for BCa, in contrast, is used in the salvage setting after surgery, for palliation, or as a part of trimodal therapy [21]. Following the receipt of RT for BCa, our study showed an increase in the incidence rate of any secondary malignancy (35.06% vs 33.38%, IRR 1.05) and a decrease in the incidence rate of secondary pelvic malignancies (11.92% RT vs 14.02% non-RT, IRR 0.85). Owing to the poor OS of patients undergoing RT for BCa, few studies have examined the effect of RT on secondary malignancy development for primary BCa [23]. Bladder RT presents technical challenges due to frequent changes in bladder volume and position, which lead to differences in the radiation doses that the bladder and nearby organs receive. While it is unexpected for our data to suggest that RT may be protective for secondary pelvic malignancy development in this population, the increased rate of any secondary malignancy highlights the carcinogenic effects of RT even though poor OS is likely modifying these results [23].

With ARC, RT is often utilized in multimodal treatment regimens as neoadjuvant therapy [24] for rectal cancer as well as primary therapy for early-stage anal cancer [25]. Data from a population-based cohort of over 13 000 patients with rectal cancer who were stratified based on radiation receipt or a lack thereof following surgical treatment showed that the 20-yr CI of secondary cancer was 16.5% in RT and 17.4% in non-RT patients. More specifically, male RT patients had a significantly lower risk of secondary PCa than their non-RT counterparts. This potential protective effect of RT in terms of secondary malignancy development after primary ARC supports the IRR of 0.94 (*p* < 0.001) from our data [26].

In UCa, RT (as EBRT or vaginal brachytherapy) is used as the primary treatment for poor surgical candidates or as adjuvant treatment for high- to intermediate-risk patients [21]. Two randomized control trials (Post-Operative Radiation Therapy in Endometrial Cancer, PORTEC-1 and PORTEC-2) examined the long-term risk of adjuvant radiation [27], [28], [29]. PORTEC-1 demonstrated a 22% rate of secondary cancers in the EBRT group compared with 16% in the no-RT group [27], [28], while PORTEC-2 demonstrated a 0.9% rate of second pelvic cancers in the EBRT group and 6.3% in the brachytherapy for a hazard ratio of 6.65 (*p* < 0.001) over a period of 10 yr [27], [28]. The results of these trials show RT as a risk factor for the development of secondary malignancies and specifically pelvic malignancies, which are consistent with our findings.

In CCa, RT is used as primary curative treatment, often for early-stage disease, with results comparable with those of surgery [21]. Matsuo et al [30] examined UCa recurrences after cervical radiation using the SEER database, showing an increasing rate of secondary UCa diagnosis after RT at 5-, 10-, and 20-yr intervals (<0.1%, 0.6%, and 1.2%, respectively), with most cases occurring 6 yr after RT [30]. These findings demonstrate the relative importance of latency period in cancers with excellent cancer-specific survival.

Unique to our study, we further analyzed secondary malignancy latency; the median time to secondary pelvic malignancy development stratified by primary disease site, SEER stage, and RT receipt demonstrated variability, particularly when evaluated in the context of OS (Supplementary Table 4 and Fig. 2). Localized BCa and CCa showed significant reductions in the median time to a secondary pelvic malignancy in RT patients compared with non-RT (23 vs 70 mo and 81 vs 131 mo, respectively). However, considering that the median OS in localized BCa was 130 mo, adjustment in therapy guidelines is likely unnecessary. However, considering that the median OS for patients with localized CCa was 378 mo, the ∼4-yr earlier development of a secondary pelvic malignancy may indicate a need for further evaluation of RT within the context of CCa management, or at least proper patient counseling.

Younger patients diagnosed with malignancies that have excellent prognosis, such as those with CCa, who are treated with RT, are more likely to live long enough to develop a second malignancy. In these patients, the development of a second malignancy may impact survival outcomes. This was confirmed by Yang et al [31] who used SEER to evaluate survival after RT in CCa and found that RT reduces survival of younger patients (<45 yr of age), those with a lower TNM stage, or those with smaller tumors. In our study, CCa patients had a higher rate of overall secondary malignancies after RT (IRR 1.80), with a significant increase in secondary pelvic malignancies between RT and non-RT patients (4.51% vs 2.62%, IRR 1.72). Our secondary analysis of latency period and survival shows a significant difference between RT and non-RT median time to a secondary pelvic malignancy, which warrants further investigation.

Other than patients with localized CCa, however, we found that for most patients with pelvic malignancies undergoing pelvic RT, the absolute rates of secondary or secondary pelvic malignancies were low. While pelvic RT increased the rates of several secondary pelvic malignancies, when put in the context of median OS and decrease in latency period, the impact of RT likely does not carry enough weight to change practice patterns. Even though the absolute risk is small, all patients receiving RT should continue to be counseled regarding the risk of a secondary malignancy following RT, with particular emphasis on patient selection in young females.

Limitations of our study include the retrospective nature of SEER with a selection bias regarding which patients can undergo RT. Radiation timing may not always have been at the time of primary malignancy diagnosis; thus, latency data are derived and may overestimate the latency period. SEER staging of localized, regional, and distant cancer provides less information than TNM staging. Our analysis did not evaluate radiation receipt based on modality. Such staging limitations and variation in radiation delivery may have had a significant impact on the reported incidence of secondary malignancies. Future studies are warranted to elucidate the impact of different forms of RT on secondary malignancy development. A further analysis of OS within the context of secondary malignancy development is critical to better understand the role of RT in the management of primary malignancies with good prognosis.

## 5 Conclusions

RT for pelvic malignancies increases the risk of developing secondary malignancies, specifically secondary pelvic malignancies. However, the absolute risk remains low for most patient populations. When put in the context of median OS and decrease in latency period, the impact of RT likely does not carry enough weight to change practice patterns. The lone exception may be patients with localized CCa, who have excellent cancer-specific survival but may experience a second pelvic malignancy nearly 4 yr earlier due to RT.

  ***Author contributions*:** Thenappan Chandrasekar had full access to all the data in the study and takes responsibility for the integrity of the data and the accuracy of the data analysis.

  *Study concept and design*: McPartland, Salib, Chandrasekar.

*Acquisition of data*: McPartland, Salib.

*Analysis and interpretation of data*: McPartland, Salib, Chandrasekar.

*Drafting of the manuscript*: McPartland, Salib.

*Critical revision of the manuscript for important intellectual content*: McPartland, Salib, Mark, Lallas, Trabulsi, Gomella, Goldberg, Den.

*Statistical analysis*: McPartland, Salib, Banks, Leiby, Chandrasekar.

*Obtaining funding*: Chandrasekar.

*Administrative, technical, or material support*: Banks, Leiby.

*Supervision*: Chandrasekar.

*Other*: None.

  ***Financial disclosures:*** Thenappan Chandrasekar certifies that all conflicts of interest, including specific financial interests and relationships and affiliations relevant to the subject matter or materials discussed in the manuscript (eg, employment/affiliation, grants or funding, consultancies, honoraria, stock ownership or options, expert testimony, royalties, or patents filed, received, or pending), are the following: None.

  ***Funding/Support and role of the sponsor*:** None.
