# Supplementary material for: Risk of Secondary Malignancies After Pelvic Radiation: A Population-based Analysis
Source: Eur Urol Open Sci. 2024 Mar 23;63:52–61. doi: 10.1016/j.euros.2024.02.013 (PMC10979055; doi:10.1016/j.euros.2024.02.013)
Supplement: Supplementary data 1 [file mmc1.docx]

**Supplementary Table 1** Patient Demographics Stratified by Primary Disease Site and Receipt of Radiation Therapy

| **Variable** | **CERVICAL CANCER** | | **PROSTATE CANCER** | | **UTERINE CANCER** | | **BLADDER CANCER** | | **RECTAL/ANAL CANCER** | |
| --- | --- | --- | --- | --- | --- | --- | --- | --- | --- | --- |
| **Radiation Receipt** | Yes | No | Yes | No | Yes | No | Yes | No | Yes | No |
| **Total** | 45736 | 42683 | 386737 | 801258 | 70913 | 178340 | 18061 | 296232 | 98366 | 160984 |
| **Sex** |  |  |  |  |  |  |  |  |  |  |
| Male | 0 | 0 | 386737 | 801258 | 0 | 0 | 12737 (71.3) | 223106 (75.3) | 56362 (57.3) | 87174 (54.2) |
| Female | 45736 | 42683 | 0 | 0 | 70913 | 178340 | 5324 (29.5) | 73126 (24.7) | 42004 (42.7) | 73810 (45.9) |
| **Age Range** |  |  |  |  |  |  |  |  |  |  |
| 20-29 years | 1563 (3.4) | 4476 (10.5) | 8 (0.0) | 31 (0.0) | 145 (0.2) | 1189(0.7) | 14 (0.1) | 1054 (0.4) | 649 (0.7) | 936 (0.6) |
| 30-39 years | 7269 (15.9) | 12177 (28.5) | 66 (0.04) | 482 (0.05) | 1319 (1.9) | 6883 (3.9) | 85 (0.5) | 3718 (1.3) | 3667 (3.7) | 3825 (2.4) |
| 40-49 years | 11159 (24.4) | 10810 (25.3) | 5407 (1.4) | 22933 (2.9) | 5484 (7.7) | 19960 (11.0) | 560 (3.1) | 14027 (4.7) | 13232 (13.5) | 13352 (8.3) |
| 50-59 years | 10484 (22.9) | 6343 (14.9) | 55280 (14.3) | 156561 (19.5) | 17596 (24.8) | 49746 (27.9) | 2002 (11.1) | 43083 (14.5) | 258882 (26.3) | 34817 (21.6) |
| 60-69 years | 7916 (17.3) | 4435 (10.4) | 151498 (39.2) | 289951 (36.2) | 24529 (34.6) | 52904 (29.7) | 4165 (23.1) | 79502 (26.8) | 27366 (27.8) | 40665 (25.3) |
| 70-79 years | 4811 (10.5) | 2574 (6.0) | 152000 (39.3) | 212939 (26.6) | 16060 (6.5) | 31057 (12.5) | 5745 (31.8) | 89550 (30.2) | 19093 (19.4) | 38416 (23.9) |
| 80+ | 2534 (5.5) | 1868 (4.4) | 22478 (5.8) | 118361 (14.8) | 5780 (8.2) | 16601 (9.3) | 5490 (30.4) | 65298 (22.0) | 8477 (8.6) | 28973 (18.0) |
| **Marital Status** |  |  |  |  |  |  |  |  |  |  |
| Unknown | 1796 (3.9) | 3411 (8.0) | 30882 (8.0) | 104977 (11.8) | 2382 (3.4) | 9081 (5.1) | 597 (3.3) | 18434 (6.2) | 3453 (3.5) | 11014 (6.8) |
| Single | 10306 (22.5) | 9976 (23.4) | 33615 (8.7) | 71794 (9.0) | 10273 (14.5) | 29182 (16.4) | 1787 (9.9) | 29032 (9.8) | 15701 (16.0) | 20780 (12.9) |
| Married | 19182(41.9) | 19923 (46.7) | 272670 (70.5) | 518346 (64.7) | 36591 (51.6) | 91854 (51.5) | 10020 (55.5) | 181456 (61.3) | 55797 (56.7) | 86533 (53.8) |
| Separated, Divorced | 5466 (12.8) | 7295(16.0) | 27792 (7.2) | 50608(6.3) | 7514 (10.6) | 18952 (10.2) | 1657 (9.2) | 23027 (7.8) | 11620 (11.8) | 14218 (8.8) |
| Widowed | 7094(15.5) | 3851 (9.0) | 21525 (5.6) | 55028 (6.9) | 14083 (19.9) | 32110 (17.4) | 3986 (21.1) | 44086 (14.9) | 11628 (11.8) | 28322 (17.6) |
| Unmarried or Domestic Partner | 63 (0.2) | 56 (0.1) | 253 (0.1) | 505 (0.1) | 70 (0.1) | 212 (0.1) | 14 (0.1) | 197 (0.1) | 167 (0.2) | 117 (0.1) |
| **Stage** |  |  |  |  |  |  |  |  |  |  |
| Localized | 11648 (25.5) | 31369 (73.5) | 223605 (57.8) | 376797 (47.0) | 38866 (54.8) | 133474 (74.8) | 3474 (19.2) | 222405 (75.1) | 28923 (29.4) | 82199 (51.1) |
| Regional | 26378 (57.7) | 4196 (9.8) | 130080 (3.4) | 52341 (6.5) | 22367 (31.5) | 18862 (10.6) | 11033 (61.1) | 45362 (15.3) | 50928 (51.8) | 36614 (22.7) |
| Distant | 6245 (13.7) | 3141 (7.4) | 10164 (2.6) | 37796 (4.7) | 7698 (10.9) | 16221 (9.1) | 2862 (15.9) | 9299 (3.1) | 13631 (13.9) | 28396 (17.6) |
| Unknown/Unstaged | 1465 (3.2) | 3977 (9.3) | 139888 (36.2) | 334324 (4.7) | 1982 (2.8) | 9783 (5.5) | 692 (3.8) | 19166 (6.5) | 4884 (5.0) | 13775 (8.6) |
| **Chemotherapy** |  |  |  |  |  |  |  |  |  |  |
| No/Unknown | 20619 (45.1) | 40269 (94.3) | 52094 (98.0) | 1125930 (99.2) | 55679 (78.5) | 160178 (89.8) | 10540 (58.4) | 255692 (86.3) | 10540 (58.4) | 255692 (86.3) |
| Yes | 25117 (54.9) | 2414 (5.7) | 3746 (1.0) | 5977 (0.8) | 15234 (21.5) | 18162 (10.2) | 7521 (41.6) | 40540 (13.7) | 7521 (41.6) | 40540 (13.7) |
| **Race** |  |  |  |  |  |  |  |  |  |  |
| Unknown | 104 (0.2) | 738 (1.7) | 3974 (1.0) | 21430 (2.7) | 151 (0.2) | 1446 (0.8) | 17 (0.1) | 2991 (1.0) | 205 (0.2) | 1913 (1.2) |
| Hispanic | 7854 (18.4) | 8660 (18.9) | 26046 (6.7) | 61437 (7.7) | 5260 (7.4) | 16654 (9.3) | 784 (4.3) | 14819 (5.0) | 9252 (4.3) | 12941 (8.0) |
| American Indian | 471 (1.0) | 376 (0.9) | 1248 (0.4) | 2958 (0.4) | 346 (0.5) | 1018 (0.6) | 56 (0.3) | 799 (0.3) | 802 (0.8) | 839 (0.5) |
| Asian | 4104 (9.0) | 3638 (8.5) | 2324 (4.4) | 52539 (4.6) | 4164 (5.9) | 12903 (7.1) | 735 (4.1) | 11112 (3.8) | 7369 (7.5) | 13664 (8.5) |
| Black | 7382 (16.1) | 5147 (12.1) | 58555 (15.1) | 107535 (13.4) | 6391 (9.0) | 15011 (8.4) | 1478 (8.2) | 14705 (5.0) | 8803 (9.0) | 15300 (9.5) |
| White | 25015 (54.7) | 24930 (58.4) | 277975 (71.9) | 571968 (71.4) | 54601 (77.0) | 131308 (73.6) | 14983 (83.0) | 251806 (85.0) | 71935 (73.1) | 116327 (72.3) |
| **Year of Diagnosis** |  |  |  |  |  |  |  |  |  |  |
| 1975-1984 | 6591 (14.4) | 5011 (11.7) | 14893 (3.9) | 49857 (6.2) | 13281 (18.7) | 15148 (8.4) | 4666 (25.8) | 26671 (9.0) | 5973 (6.0) | 24004 (14.9) |
| 1985-1994 | 6460 (14.1) | 6758 (15.8) | 43824 (11.3) | 108758 (13.6) | 9546 (13.5) | 19557 (11.1) | 2524 (14.0) | 38577 (13.0) | 10994 (11.2) | 23901 (14.9) |
| 1995-2004 | 12602 (27.6) | 12956 (30.4) | 131719 (34.1) | 236703 (29.5) | 15809 (22.3) | 45615 (25.6) | 3808 (21.1) | 83398 (28.2) | 27444 (27.9) | 43871 (27.3) |
| 2005-2016 | 20083 (43.9) | 17958 (42.1) | 196301 (50.8) | 405940 (50.7) | 32277 (45.5) | 980020 (55.0) | 7063 (39.1) | 147586 (49.8) | 54055 (55.0) | 69208 (43.0) |

**Supplementary Table 2**: Cumulative Incidence of ANY Secondary Malignancy Stratified by Primary Disease Site, RT receipt, Time Frame, and SEER Stage

| PRIMARY DISEASE SITE | SEER Stage | Time frame | No Radiation | | Radiation | | Difference 95% CI |
| --- | --- | --- | --- | --- | --- | --- | --- |
|  |  |  | No. at risk | Cumulative Incidence 95% CI | No. at risk | Cumulative Incidence 95% CI |  |
| Cervical | Localized | 5 years | 22312 | 4.26(4.03-4.50) | 6520 | 5.99 (5.55-64.5) | 1.73 (1.22-2.24) * |
|  |  | 10 years | 16170 | 6.99 (6.68-7.30) | 4155 | 10.03 (9.43-10.64) | 3.04 (2.36-3.72) * |
|  |  | 15 years | 10671 | 9.80 (9.41-10.20) | 2490 | 13.50 (12.77-14.24) | 3.70 (2.86-4.53) * |
|  | Regional | 5 years | 1500 | 4.51 (3.88-5.21) | 9795 | 5.44 (5.16-5.74) | 0.93 (0.21-1.65) * |
|  |  | 10 years | 942 | 6.91 (6.10-7.80) | 5496 | 8.62 (8.25-9.01) | 1.71 (0.78-2.64) * |
|  |  | 15 years | 578 | 8.11 (7.18-10.68) | 2785 | 11.14 (10.68-11.61) | 3.03 (1.96-4.10) * |
|  | Distant | 5 years | 162 | 1.91 (1.45-2.46) | 758 | 3.03 (2.61-3.51) | 1.12 (0.45-1.80) * |
|  |  | 10 years | 56 | 2.57 (1.98-3.28) | 370 | 4.22 (3.68-4.81) | 1.66 (0.80-2.51) * |
|  |  | 15 years | - | - | 180 | 4.96 (4.33-5.63) | - |
| Prostate | Localized | 5 years | 219124 | 6.77(6.68-6.86) | 144775 | 8.04 (7.92-8.17) | 1.28 (1.13-1.42) * |
|  |  | 10 years | 94176 | 12.18 (12.05-12.31) | 65642 | 15.39 (15.21-15.57) | 3.21 (2.99-3.43) * |
|  |  | 15 years | 22264 | 16.59 (16.41-16.78) | 14120 | 21.21 (20.97-21.46) | 4.62 (4.31-4.92) * |
|  | Regional | 5 years | 32396 | 6.01 (5.79-6.23) | 7430 | 7.44 (6.95-7.95) | 1.43 (0.89-1.98) * |
|  |  | 10 years | 16059 | 11.60 (11.26-11.94) | 3364 | 13.04 (12.34-13.76) | 1.44 (0.66-2.22) * |
|  |  | 15 years | 4602 | 16.92 (16.43-17.41) | 963 | 17.93 (16.98-18.91) | 1.01 (-0.07-2.09) |
|  | Distant | 5 years | 4313 | 5.52 (5.26-5.77) | 1272 | 5.85 (5.35-6.37) | 0.33 (-0.24-0.90) |
|  |  | 10 years | 978 | 6.86 (6.56-7.16) | 346 | 7.90 (7.28-8.55) | 1.04 (0.34-1.75) * |
|  |  | 15 years | 193 | 7.36 (7.04-7.69) | 76 | 9.19 (8.44-9.98) | 1.83 (0.99-2.67) * |
| Uterine | Localized | 5 years | 86591 | 2.83 (2.73-2.93) | 24510 | 3.27 (3.08-3.46) | 0.43 (0.21-0.65) * |
|  |  | 10 years | 54269 | 7.65 (7.48-7.82) | 16444 | 7.70(7.40-8.00) | 0.05 (-0.03-0.04) |
|  |  | 15 years | 30323 | 13.57 (13.32-13.82) | 10552 | 12.79 (12.39-13.19) | -1.12 (-1.65- -0.70) * |
|  | Regional | 5 years | 6752 | 4.54 (4.22-4.88) | 10630 | 3.67 (3.41-3-3.95) | -0.88 (-1.30- -0.45) * |
|  |  | 10 years | 3162 | 9.16 (8.65-9.69) | 5857 | 8.59 (8.16-9.04) | -0.06 (-0.74-0.62) |
|  |  | 15 years | 1401 | 13.10 (12.41-13.80) | 2774 | 14.17 (13.56-14.79) | 1.07 (0.14-2.00) * |
|  | Distant | 5 years | 1835 | 2.88 (2.60-3.17) | 1834 | 3.06 (2.67-3.49) | 0.18 (-0.31-0.68) |
|  |  | 10 years | 836 | 4.06 (3.72-4.43) | 994 | 4.91 (4.38-5.48) | 0.84 (0.19-1.50) * |
|  |  | 15 years | 402 | 5.09 (4.67-5.54) | 538 | 7.32 (6.62-8.06) | 2.23 (1.39-3.07) * |
| Bladder | Localized | 5 years | 116016 | 14.38 (14.23-14.53) | 916 | 11.86 (10.76-13.01) | -2.52(-3.66- -1.38) * |
|  |  | 10 years | 58086 | 21.73 (21.54-21.92) | 455 | 15.25 (14.00-16.55) | -6.47 (-7.76- -5.18) * |
|  |  | 15 years | 25597 | 26.40 (26.18-26.62) | 253 | 16.76 (15.45-18.12) | -9.64 (-10.99- -8.28) * |
|  | Regional | 5 years | 10724 | 16.23 (15.89-16.58) | 1963 | 8.23 (7.71-8.77) | -8.00(-8.36- -7.37) * |
|  |  | 10 years | 5155 | 18.69 (18.31-19.07) | 847 | 10.17 (9.59-10.78) | -8.52 (-9.22- -8.36) * |
|  |  | 15 years | 2176 | 20.28 (19.88-20.68) | 407 | 11.17 (10.54-11.81) | -9.11 (-9.86- -8.37) * |
|  | Distant | 5 years | 260 | 4.75 (4.32-5.20) | 79 | 2.94 (2.35-5.20) | -1.81 (-2.58- -1.03) * |
|  |  | 10 years | - | - | - | - | - |
|  |  | 15 years | 40 | 5.26 (4.79-5.75) | - | - | - |
| Rectal and Anal | Localized | 5 years | 44960 | 8.80(8.60-9.01) | 14329 | 7.74 (7.42-8.07) | -1.06 (-1.45—0.62) * |
|  |  | 10 years | 24666 | 14.35 (14.08-14.62) | 6870 | 13.21 (12.76-13.66) | -1.15 (-1.67 - -0.62) * |
|  |  | 15 years | 11750 | 18.42 (18.10-18.74) | 2563 | 17.42 (16.85-17.99) | -1.81 (-2.60 - -1.03) * |
|  | Regional | 5 years | 13173 | 7.06 (6.79-7.34) | 20175 | 6.70 (6.47-6.94) | -0.36 (-0.72- 0.00) |
|  |  | 10 years | 6506 | 10.08 (9.75-10.42) | 9143 | 10.73 (10.42-11.05) | 0.65 (0.19-1.11) * |
|  |  | 15 years | 3035 | 12.29 (11.91-12.68) | 3788 | 13.84 (13.45-14.24) | 1.55 (1.00-2.10) * |
|  | Distant | 5 years | 1234 | 2.19 (2.02-2.38) | 1353 | 3.37 (3.06-3.70) | 1.18 (0.81-1.54) * |
|  |  | 10 years | 363 | 2.46 (2.27-2.66) | 397 | 4.53 (4.14-4.95) | 2.07 (1.62-2.52) * |
|  |  | 15 years | 131 | 2.65 (2.45-2.87) | 127 | 5.40 (4.91-5.91) | 2.74 (2.19-3.29) * |

*= denotes statistically significant confidence interval

**Supplementary Table 3**: Cumulative Incidence of Secondary PELVIC Malignancy Stratified by Primary Disease Site, RT receipt, Time Frame, and SEER Stage

| PRIMARY DISEASE SITE | SEER Stage | Time frame | No Radiation | | Radiation | | Difference 95% CI |
| --- | --- | --- | --- | --- | --- | --- | --- |
|  |  |  | No. at risk | Cumulative Incidence 95% CI | No. at risk | Cumulative Incidence 95% CI |  |
| Cervical | Localized | 5 years | 22839 | 1.90 (1.75-2.07) | 6746 | 1.94 (1.69-2.22) | 0.04 (-0.27-0.34) |
|  |  | 10 years | 16760 | 2.53 (2.35-2.73) | 4394 | 2.93 (2.61-3.29) | 0.40(0.02-0.79) * |
|  |  | 15 years | 11218 | 3.16 (2.94-3.39) | 2674 | 3.98 (3.57-4.42) | 0.82 (0.34-1.29) * |
|  | Regional | 5 years | 1542 | 2.00 (1.59-2.49) | 10185 | 1.67 (1.51-1.84) | -0.33 (-0.80-0.14) |
|  |  | 10 years | 998 | 2.49 (2.02-3.04) | 5832 | 2.51 (2.30-2.73) | 0.01 (-0.54-0.57) |
|  |  | 15 years | 617 | 2.85 (2.32-3.47) | 2993 | 3.30 (3.04-3.58) | 0.45 (-0.18-1.09) |
|  | Distant | 5 years | 168 | 0.89 (0.59-1.30) | 789 | 0.99 (0.76-1.28) | 0.10 (-0.38-0.58) |
|  |  | 10 years | 61 | 1.03 (0.69-1.50) | 397 | 1.33 (1.03-1.68) | 0.30 (-1.09-1.68) |
|  |  | 15 years | - | - | 195 | 1.70 (1.32-2.15) | - |
| Prostate | Localized | 5 years | 228008 | 1.06 (1.03-1.10) | 151430 | 1.46 (1.41-1.52) | 0.40 (0.33-0.46) * |
|  |  | 10 years | 100848 | 1.92 (1.86-1.97) | 71035 | 2.97 (2.89-3.06) | 1.06 (0.96-1.16) * |
|  |  | 15 years | 24462 | 2.63 (2.54-2.71) | 15665 | 4.69 (4.56-4.83) | 2.07 (1.91-2.23) * |
|  | Regional | 5 years | 33546 | 1.07 (0.98-1.17) | 7760 | 1.43 (1.21-1.67) | 0.35 (0.11-0.60) * |
|  |  | 10 years | 17147 | 1.90 (1.76-2.05) | 3588 | 2.38 (2.07-2.71) | 0.48 (0.13-0.83) * |
|  |  | 15 years | 5049 | 2.64 (2.45-2.85) | 1049 | 3.68 (3.20-4.20) | 1.03 (0.49-1.57) * |
|  | Distant | 5 years | 5627 | 1.12 (1.01-1.24) | 1735 | 1.06 (0.86-1.30) | -0.06(-0.31-0.19) |
|  |  | 10 years | 1387 | 1.33 (1.20-1.47) | 521 | 1.32 (1.08-1.61) | 0.00(-0.30-0.29) |
|  |  | 15 years | 310 | 1.44 (1.30-1.60) | 125 | 1.67 (1.34-2.03) | 0.22 (-0.15-0.60) |
| Uterine | Localized | 5 years | 86217 | 1.38 (1.32-1.45) | 24407 | 1.34 (1.23-1.47) | -0.04 (-0.17-0.10) |
|  |  | 10 years | 53887 | 1.91 (1.83-1.99) | 16311 | 1.90 (1.76-2.06) | 0.00 (-0.18-0.16) |
|  |  | 15 years | 30017 | 2.34 (2.24-2.44) | 10378 | 2.55 (2.37-2.74) | 0.40 (0.19-0.61) |
|  | Regional | 5 years | 6698 | 2.20 (1.99-2.43) | 10578 | 1.52 (1.35-1.69) | -0.68 (-0.95- -0.41) * |
|  |  | 10 years | 3127 | 2.71 (2.46-2.98) | 5791 | 2.12 (1.92-2.35) | -0.59 (-0.93- -0.25) * |
|  |  | 15 years | 1358 | 3.00 (2.71-3.31) | 2725 | 2.97 (2.68-3.27) | -0.03 (-0.45-0.39) |
|  | Distant | 5 years | 1846 | 0.95 (0.81-1.12) | 1837 | 0.95 (0.74-1.20) | 0.00 (-0.27-0.27) |
|  |  | 10 years | 835 | 1.12 (0.95-1.31) | 986 | 1.32 (1.06-1.63) | 0.20 (-0.13-0.53) |
|  |  | 15 years | 397 | 1.22 (1.03-1.43) | 529 | 1.46 (1.17-1.79) | 0.24 (-0.13-0.53) |
| Bladder | Localized | 5 years | 123697 | 6.67 (6.57-6.78) | 1012 | 4.86 (4.15-5.65) | -1.81 (-2.57- -1.05) * |
|  |  | 10 years | 63773 | 9.60 (9.46-9.73) | 514 | 5.68 (4.90-6.54) | -3.92 (-4.75- -3.08) * |
|  |  | 15 years | 28696 | 11.45 (11.29-11.61) | 292 | 6.04 (5.22-6.94) | -5.41(-6.28- -4.52) * |
|  | Regional | 5 years | 11595 | 11.27 (10.98-11.57) | 2107 | 3.22 (2.90-3.57) | -8.05 (-8.50- -7.60) * |
|  |  | 10 years | 5664 | 11.80 (11.49-12.11) | 931 | 3.64 (3.29-4.02) | -8.16 (-8.63 - -7.68) * |
|  |  | 15 years | 2418 | 12.14 (11.82-12.45) | 461 | 3.87 (3.49-4.28) | -8.26 (-8.76- -7.76) * |
|  | Distant | 5 years | 282 | 3.04 (2.70-3.41) | 83 | 1.54 (1.13-2.06) | -1.49 (-2.08- -0.91) * |
|  |  | 10 years | 126 | 3.15 (2.80-3.53) | - | - | - |
|  |  | 15 years | 45 | 3.25 (2.89-3.65) | - | - | - |
| Rectal and Anal | Localized | 5 years | 47618 | 3.30 (3.18-3.44) | 15205 | 2.05 (1.89-2.23) | -1.25 (-1.47- -1.03) * |
|  |  | 10 years | 26858 | 5.38 (5.21-5.55) | 7464 | 3.87 (3.61-4.14) | -1.51 (-1.82- -1.19) * |
|  |  | 15 years | 13083 | 6.96 (6.75-7.18) | 2849 | 5.31 (4.96-5.66) | -1.66 (-2.07- -1.24) * |
|  | Regional | 5 years | 14100 | 2.38 (2.22-2.55) | 21384 | 2.02 (1.89-2.15) | -0.36 (-0.57- -0.15) * |
|  |  | 10 years | 7129 | 3.31 (3.11-3.51) | 9988 | 3.19 (3.01-3.37) | -0.12 (-0.39- 0.15) |
|  |  | 15 years | 3414 | 4.10 (3.87-4.34) | 4205 | 4.31 (4.07-4.55) | 0.21 (-0.12-0.55) |
|  | Distant | 5 years | 1331 | 0.72 (0.62-0.83) | 1436 | 0.90 (0.74-1.07) | 0.92 (-0.10-0.29) |
|  |  | 10 years | 405 | 0.80 (0.69-0.92) | 444 | 1.26 (1.05-1.50) | 0.46 (-1.12-2.04) |
|  |  | 15 years | 150 | 0.86 (0.74-1.00) | 149 | 1.62 (1.32-1.95) | 0.75 (0.41-1.09) * |

*= denotes statistically significant confidence interval

**Supplementary Table 4**: Median time to Secondary PELVIC Malignancy Stratified by Primary Disease Site, RT receipt, and SEER Stage

| PRIMARY DISEASE SITE | SEER Stage | No Radiation | | Radiation | | Difference 95% CI |
| --- | --- | --- | --- | --- | --- | --- |
|  |  | No. at risk | Median time to 2^nd^ pelvic malignancy (months) | No. at risk | Median time to 2^nd^ pelvic malignancy (months) |  |
| Cervical | Localized | 22839 | 131.00 | 6746 | 81.00 | -50.00 (-53.72- -46.28) * |
|  | Regional | 1542 | 27.00 | 10185 | 37.00 | 10.00(7.61-12.39) * |
|  | Distant | 168 | 4.00 | 789 | 12.00 | 8.00 (7.77-8.23) * |
| Prostate | Localized | 228008 | 76.00 | 151430 | 88.00 | 12.00 (11.69-12.31) * |
|  | Regional | 33546 | 85.00 | 7760 | 76.00 | -9.00 (-10.95- -7.05) * |
|  | Distant | 5627 | 19.00 | 1735 | 20.00 | 1.00 (0.10-1.90) * |
| Uterine | Localized | 86217 | 93.00 | 24407 | 94.00 | 1.00 (-0.94-2.93) |
|  | Regional | 6698 | 35.00 | 10578 | 54.00 | 19.00 (17.39-20.61) * |
|  | Distant | 1846 | 10.00 | 1837 | 19.00 | 9.00 (7.82-10.18) * |
| Bladder | Localized | 123697 | 70.00 | 1012 | 23.00 | -47.00 (-48.61- -45.39) * |
|  | Regional | 11595 | 17.00 | 2107 | 17.00 | 0(-1.15-1.15) |
|  | Distant | 282 | 4 | 83 | 6 | 2 * |
| Rectal and Anal | Localized | 47618 | 76.00 | 15205 | 64.00 | -12.00 (-13.58- -10.42) * |
|  | Regional | 14100 | 40.00 | 21384 | 47.00 | 7.00 (5.92- -8.08) |
|  | Distant | 1331 | 9.00 | 1436 | 15.00 | 6 (5.72-6.27) * |

*= denotes statistically significant confidence interval
